# Normative beliefs and values that shape care-seeking behaviours for skilled birth attendance (SBA) during birthing by mothers in Africa: a scoping review protocol

**DOI:** 10.1186/s13643-021-01629-1

**Published:** 2021-03-29

**Authors:** Choolwe Jacobs, Adnan A. Hyder

**Affiliations:** 1 School of Public Health, University of Zambia, Nationalist Road, Box 50110, Lusaka, Zambia; 2 Berman Institute of Bioethics, Johns Hopkins University, Baltimore, MD USA; 3 Milken Institute School of Public Health, George Washington University, Washington, DC USA

**Keywords:** Skilled birth attendance, Beliefs, Values, Africa

## Abstract

**Background:**

**S**killed birth attendance (SBA) during delivery has been associated with improved maternal health outcome. However, low utilisation of SBA during childbirth has continued in many developing countries including Zambia. The proposed scoping review aims to map literature on beliefs and values and how mothers are influenced by relational normative motivations in utilisation of SBAs in health facilities.

**Methods:**

We designed and registered a study protocol for a scoping review. Literature searches will be conducted in multiple electronic databases (from January 2000 onwards), including PubMed, EMBASE, Scopus and Web of Science. Grey literature will be identified through searching dissertation databases, Google Scholar, EBSCOhost and ResearchGate. Keyword searches will be used to identify articles. Only articles published in English, related on beliefs and values surrounding childbirth, and on perceptions towards facility delivery or skilled health care providers will be considered eligible for inclusion. Two reviewers will independently screen eligible titles, abstracts and full articles with a third reviewer to help resolve any disputes. The study methodological quality (or bias) will be appraised using the Mixed Method Appraisal Tool. A narrative summary of findings will be conducted. We will employ NVIVO version 10 software package to extract the relevant outcomes from the included articles using content thematic analysis. This protocol is registered with the Open Science Framework (osf.io/9gn76).

**Discussion:**

Understanding how individual mother’s health seeking behaviours for SBA and those close to them are influenced by their beliefs and values is critical to informing health systems on the possible ‘hidden’ barriers and facilitators to utilisation of SBA in public health facilities. The review will complement evidence base on normative beliefs and values shaping care-seeking behaviours for skilled birth attendance by mothers in Africa.

**Supplementary Information:**

The online version contains supplementary material available at 10.1186/s13643-021-01629-1.

## Background

Utilisation and access to quality facility-based services by skilled birth attendants (SBAs) during pregnancy, delivery, and post-delivery has been associated with improved maternal health outcomes [1, 2]. However, low utilisation of care by SBAs during childbirth has continued in many developing countries.

Existing evidence reveals that individual and contextual factors, including health system factors, influence mothers’ care-seeking behaviours for skilled birth attendance in health facilities during childbirth [3, 4]. Health system factors that hinder care seeking for childbirth have been reported to include limited availability of health care workers, lack of equipment and supplies [5, 6], and the attitude [7] and practices of health care workers, such as disrespectful and abusive care [8]. Individual level factors, such as the mother’s age [9], marital status [10], level of education, economic status of the household [11, 12] and cultural and religious beliefs [13–16] have also been reported as negative predictors to care-seeking behaviours of mothers. The location of services available is another important contextual factor hindering utilisation of SBA for childbirth from the health facilities [17].

Some studies have reported that although some mothers are willing to seek care for childbirth [18] and would prefer to give birth in a health centre with a skilled health care provider [19], the perceptions of care provided by skilled attendants influences the intention and decision to seek care [20, 21]. This becomes one of the contributing factors to the existing disparities to access and utilisation of care from SBAs during childbirth.

The growing need for health systems to ensure better maternal and infant health outcomes for populations requires continued exploration of possible hidden barriers using diverse approaches that inform policy. An understanding of the beliefs and values and how individual mothers are influenced by normative motivations is critical to informing public health policy on the possible ‘hidden’ barriers and facilitators to utilisation of SBA in public health facilities. Normative motivations are concerned with the individual’s view of what is morally right and wrong [22]. These motivations involve doing the right thing just because it is right, and not as a means to avoid punishment or gain social rewards. Down and colleagues (2018) argue that the care of mothers during childbirth should be designed to fulfil women’s personal and socio-cultural beliefs and expectations [23]. Therefore, studies focusing on mothers’ normative values and beliefs and how these shape their decisions to seek care from a skilled birth attendant during childbirth need to be explored. However, the extent of the evidence on mothers’ normative values and beliefs and how these values may be shaping their decisions and motivation to access care by a skilled birth attendant is limited. Therefore, this scoping review aims at mapping and documenting existing literature on normative beliefs and values that shape care-seeking behaviours for SBA among mothers in Africa. We hope that the findings from this review will highlight gaps in the existing literature and form a basis for refining research questions for future interventions.

## Methods

### Search strategy

A systematic search, selection, and synthesis of existing literature will be used through a scoping review methodology in order to answer the research question. Scoping reviews are a comprehensive and rigorous method aimed at rapidly mapping key concepts underpinning a research area. This review protocol has been registered within the Open Science Framework database (registration number: osf.io/9gn76) and is being reported in accordance with the reporting guidance provided in the Preferred Reporting Items for Systematic Reviews and Meta-Analyses Protocols (PRISMA-P) statement [24] (see checklist in Additional file 1). The scoping review methodology will be conducted in accordance with the framework proposed by Arksey and O’Malley [25], which has been further developed by Levac et al. [26].

## Identifying the research question

Our research question is: What is the evidence of normative beliefs and values that shape care-seeking behaviours for SBA by mothers in Africa*?*

We have used the Population—Concept—Context (PCC) approach recommended by the Joanna Briggs Institute for scoping reviews [27] to construct a clear and meaningful research question for a scoping review.

## Identifying relevant studies

The primary studies that have a clear empirical base utilising qualitative, quantitative and mixed methods published in peer-reviewed journals will be sourced based on a structured search of electronic databases (from January 2000 onwards): PubMed, EMBASE, Scopus and Web of Science. The secondary source of potentially relevant material will be a search of dissertation databases, Google Scholar, EBSCOhost and ResearchGate. Not only references of included documents but also key journals, thesis and dissertations will be hand-searched to identify any additional evidence sources. We will also search reference lists for relevant articles and studies. Review articles will be excluded. Medical Subject Headings (MeSH) terms will be included during the search for relevant articles. The search strategy will be designed by a research librarian and peer reviewed. The search terms will include *perception, expectation, experience, mothers, women, normative belief, culture, value, norm, tradition, utilization, utilisation, uptake, maternity, delivery, childbirth, sub-Sahara, Africa developing and low- and middle-income country.* We will also use Boolean terms, AND and OR to separate the keywords. The search strategy will be adapted for each database. For each search conducted, we will document in detail the date of search, the search engine and the number of publications retrieved.

## Study selection

Identification of appropriate and relevant articles will be guided by selection criteria.

## Eligibility criteria

### Inclusion criteria

- Focus on women’s beliefs and values surrounding childbirth
- Report on experiences and perceptions towards facility delivery or skilled health care providers
- Published from January 2000 onwards
- Studies conducted in Africa

### Exclusion criteria

We will exclude the following articles:

- Studies with no evidence of mother’s values, beliefs or norms surrounding childbirth
- Studies not focused on childbirth or delivery services
- Studies not freely available in full text

## Screening and selection process

The search strategies will be piloted to check the appropriateness of the selected databases and key words. All the articles retrieved from the databases will be screened for eligibility through the following stages. In the first stage, the principle investigator (PI) will screen the all the titles for eligibility, and articles that meet the eligibility criteria will be exported to endnote, a referencing software (version 7) used to store and organize citation information. All the titles that are not eligible will be excluded and all the duplicates deleted. In the second stage, the PI and two independent trained reviewers will independently (in parallel) screen the abstracts from the retrieved titles to include only eligible articles. In the last stage, the PI and two independent reviewers shall read through the full articles for eligibility. At every stage, any differences in the outputs will be resolved through a discussion and a consensus will be derived. Reasons for exclusion of articles at each stage will be documented. A flow chart showing details of studies included and excluded at each stage of the study selection process will be provided.

## Charting the data

An analytical method will be used to extract the background information and process oriented information of each included study. A data charting form will be developed and piloted. The following variables for which data will be sought in order to answer the question will be determined and will include author(s) names, date of publication, country of publication study title, journal full reference, aims or research question, participant characteristics, sampling method, study design, theoretical background, data collection, data analysis and most relevant findings conclusions and comments. The piloting forms will be done by independent reviewers in duplicate [28]. This will be done by ensuring that the reviewers independently conduct a trial data extraction and later discuss as a team to ensure that the approach is in line with the objective. To improve the applicability, consistency and quality of the chart, a continued review through an iterative process, which involves reading and re-reading the summaries, will be ensured. This process will entail continuous editing of the extraction form throughout the duration of the study.

### Collating, summarising, and reporting the results

This study is aimed at mapping the existing evidence and to summarise the findings as presented across articles. The data from the data collection process will be quantitatively summarised using a simple numerical count and qualitatively drawn on the descriptive-analytical method using thematic analysis and visual representations (including diagrams). The quantitative results will be presented in a table format, followed by a narrative section containing the main theories. A narrative account of all the data extracted from the studies that meet the inclusion criteria will be analysed using the thematic content analysis. Data relating to the beliefs and values of mothers or women, in regards to childbirth by skilled health care providers in Africa, will be coded using NVivo version 10 [29]. Emerging themes will be identified, coded, and categorised into major themes. The resulting themes will then be synthesised, summarised and discussed in relation to the objective of the study and its implications on ethical practice, policy and future research.

## Quality appraisal

To determine the methodological quality of the studies to be included, we will use the mixed method appraisal tool (MMAT) version 2018 [30]. The MMAT tool will help us determine the quality of the studies by examining the aptness of the aim of the study and the adequacy of the methodology, study design, participant recruitment, data collection, data analysis, presentation of findings and authors’ discussions and conclusions. A scoring matrix in the MMAT tool will be used to independently appraise the quality of the studies, and the results from the scoring will be used to determine the overall quality of the article.

## Discussion

Efforts to identify drivers to non-use of SBA among women should consider different and additional dimensions including normative and ethical inquiry [31, 32]. Understanding the beliefs and values of mothers and others close to them, and how individual mothers are influenced by these beliefs and values, is critical to informing public health systems on the possible ‘hidden’ barriers and facilitators to utilisation of SBA in public health facilities. The findings from this review may inform public health and clinical practice and guide the design of strategies that respond to the beliefs and values of mothers, in efforts to achieve Universal Health Coverage for maternal health care interventions in developing countries.

Studies with no evidence of mothers’ beliefs and values including perceptions and experiences surround child birth will be excluded because the focus of this review is on normative beliefs and values that shape care-seeking behaviours for SBA by mothers in Africa. This scoping review will be limited to articles presenting findings from Africa because of the similar health issues. Specifically, this study also focuses on when the views of women in Africa.

This scoping review may have limitations worth noting. Firstly, limitations in this scoping review may arise due to the focus on mapping the breadth of studies instead of the depth of information. However, the chosen methodology is appropriate in addressing the review questions. Secondly, whilst this study by drawing on mixed methods will draw on rich information for the objective, there may be difficulty arriving at overall meaningful summary measures across heterogeneous studies.

Nevertheless, this scoping review will be critical to uncover the breadth and extent of research that exists in field. The findings of the review may be of interest to researchers by highlighting research gaps that may need further investigation. Further, implementers and policymakers involved in interventions aimed at improving SBA may be informed by the evidence from the review. Finally, the review of findings from studies in Africa will be of interest to informing health systems on some demand side barriers and facilitators to utilisation of SBA in public health facilities. The findings of this scoping review study will be published in a peer-reviewed journal.

## Supplementary Information

**Additional file 1.** PRISMA-P (Preferred Reporting Items for Systematic review and Meta-Analysis Protocols) 2015 checklist.

**Additional file 2.** PUBMED Search Strategy Summary - Date; 12^TH^ November 2020.

## Data Availability

Further details of the protocol are available from the corresponding author on reasonable request.

## Abbreviations

SBA: Skilled birth attendance
PCC: Population, Concept, Context
