# Supplementary material for: Normative beliefs and values that shape care-seeking behaviours for skilled birth attendance (SBA) during birthing by mothers in Africa: a scoping review protocol
Source: Syst Rev. 2021 Mar 29;10:87. doi: 10.1186/s13643-021-01629-1 (PMC8006368; doi:10.1186/s13643-021-01629-1)
Supplement: Supplementary file 2 — Additional file 2. PUBMED Search Strategy Summary - Date; 12TH November 2020. [file 13643_2021_1629_MOESM2_ESM.docx]

FILE 2: PUBMED SEARCH STRATEGY SUMMARY - DATE; 12^TH^ NOVEMBER 2020

| Search number | Query | Filters | Results | Time |
| --- | --- | --- | --- | --- |
| 12 | (((((#1) AND (#2)) AND (#3)) AND (#4)) AND (#5)) AND (#6) | Abstract, Full text, English, from 2000/1/1 - 2020/12/31 | 72 | 01:18:51 |
| 11 | (((((#1) AND (#2)) AND (#3)) AND (#4)) AND (#5)) AND (#6) | Abstract, English, from 2000/1/1 - 2020/12/31 | 76 | 01:13:50 |
| 10 | (((((#1) AND (#2)) AND (#3)) AND (#4)) AND (#5)) AND (#6) | English, from 2000/1/1 - 2020/12/31 | 76 | 01:13:39 |
| 8 | (((((#1) AND (#2)) AND (#3)) AND (#4)) AND (#5)) AND (#6) | English | 80 | 01:12:20 |
| 9 | (((((#1) AND (#2)) AND (#3)) AND (#4)) AND (#5)) AND (#6) | Abstract, English | 80 | 01:12:09 |
| 7 | (((((#1) AND (#2)) AND (#3)) AND (#4)) AND (#5)) AND (#6) |  | 80 | 01:11:44 |
| 6 | (((africa) OR (sub sahara africa)) OR (developing country)) OR (low income country) |  | 503,742 | 01:10:31 |
| 5 | ((child birth[MeSH Terms]) OR (delivery[MeSH Terms])) OR (giving birth[MeSH Terms]) |  | 93,928 | 01:09:17 |
| 4 | ((skilled birth attendants) OR (trained midwife)) OR (specialised birth attendant) |  | 27,852 | 01:08:16 |
| 3 | (((perception) OR (expectation)) OR (motivation)) OR (experience) |  | 2,137,596 | 01:07:08 |
| 2 | ((((normative beliefs) OR (beliefs)) OR (values)) OR (culture)) OR (custom) |  | 4,171,474 | 01:06:11 |
| 1 | ((mothers[MeSH Terms]) OR (women[MeSH Terms])) OR (female[MeSH Terms]) |  | 8,831,885 | 01:04:53 |
